# A cross-sectional study of HIV and syphilis infections among male students who have sex with men (MSM) in northeast China: implications for implementing HIV screening and intervention programs

**DOI:** 10.1186/1471-2458-11-287

**Published:** 2011-05-10

**Authors:** Jun-Jie Xu, Kathleen Heather Reilly, Chun-Ming Lu, Ning Ma, Min Zhang, Zhen-Xing Chu, Jun-Jie Wang, Ke Yun, Hong Shang

**Affiliations:** 1 From Key Laboratory of AIDS Immunology of Ministry of Health, Department of Laboratory Medicine, No.1 Hospital of China Medical University, Shenyang 110001, China; 2 National Center for AIDS/STD Control and Prevention, Chinese Center for Disease Control and Prevention(CDC), Beijing, China; 3 Tulane University Health Sciences Center, School of Public Health and Tropical Medicine, New Orleans, LA, USA; 4 Liaoning Provincial CDC, Shenyang, China

**Keywords:** HIV, syphilis, China, men who have sex with men (MSM), high school students, college students

## Abstract

**Background:**

China has 76.2 million high school and college students, in which the number of reported HIV/AIDS cases is increasing rapidly. Most of these cases are attributed to male-to-male sexual contact. Few studies have explored HIV prevalence and behavioural characteristics of Chinese male students who have sex with men (MSM).

**Methods:**

A cross-sectional study of MSM high school and college students in Liaoning Province was conducted. Data were collected through face-to-face interviews and blood specimens were obtained and tested for HIV and syphilis.

**Results:**

There were 436 eligible participants. HIV and syphilis prevalence was 3.0% and 5.0%, respectively. In multivariate analysis, sexual orientation known by family members (OR: 7.3; 95% CI: 1.5-34.6), HIV/AIDS information obtained from clinical doctors (OR: 6.7; 95% CI: 1.7-25.9), HIV/AIDS information obtained through free educational services and materials such as voluntary counseling and testing (VCT) and condom distribution services (OR: 0.2; 95% CI: 0.4-1.0), inconsistent condom use (OR: 5.7; 95%: 1.3-25.3), sexual partner experienced anal bleeding after insertive anal intercourse (OR: 6.8; 95% CI: 1.6-28.4), and history of illegal drug use (OR: 18.9; 95% CI: 2.2-165.3) were found to be significantly associated with HIV infection.

**Conclusions:**

Greater effort should be made towards stemming the HIV and syphilis epidemics among Chinese student MSM. Immediate screening and comprehensive interventions towards student MSM should be implemented in order to curb the spread of HIV. Family and school-based interventions should be considered to target this educated, yet vulnerable, population.

## Background

Acquired Immune Deficiency Syndrome (AIDS) is currently one of the leading causes of death among 15 to 24-year adolescent and young adults [1]. Reducing adolescent sexual risk behaviour has become an international public health priority [1]. In China, MSM accounted for 12.2% of new HIV cases in 2007 [2], while in 2009 the proportion increased to 32.5% of all new HIV cases [3]. Several large cities in China have found a steady increase in HIV prevalence among MSM [4-7]. It is important for China to curb the spread of the HIV epidemic among this vulnerable population. Several recent studies of Chinese MSM found that between 27.9%-72.3% of MSM study participants were college educated [8,9], which is significantly higher than the percentage of higher education among other high-risk groups in China including female sex workers (6.4-7.1%) [10,11] and injection drug users (0.0-8.1%) [12,13]. While Chinese students are generally not considered to be at high risk for HIV [2], only a few studies with small samples sizes have been conducted to analyze the risk behaviours for HIV infection among Chinese student MSM [14-16].

China has the largest number of college students in the world. By the end of 2009, there were an estimated 46.4 million high school students and 29.8 million college students in China, approximately half of whom are male [17]. Chinese studies have found that 3.7%-10.3% [16,18] of male college students reported ever having at least one episode of sexual intercourse with another male. Those students who reported homosexual behaviours had more sexual partners and higher proportions of unprotected sex behaviour compared with non-MSM male students [16].

Liaoning Province is the economic and commercial hub of northeast China with one of the largest numbers of college and high school students. Recent surveys of Liaoning MSM found a significantly higher prevalence of syphilis compared with Beijing MSM (25.4% vs.11.2%, respectively) [8,19] and syphilis infection was independent risk factor for both HIV infection and incidence [7,8,19]. Syphilis prevalence appears to be rapidly increasing among Chinese MSM [6,7]. Syphilis infection is a biomarker for unprotected sex, and may facilitate the acquisition of HIV [20].

Recently Beijing, Hefei, Chongqing, Hangzhou Cities have detected HIV infections among college and high school students [15,21-23]. In Hangzhou city, students accounted for 3.7% of newly reported HIV cases between January 1, 2009 to May 31, 2010, and the prevalence of HIV among college students reached 0.4% (8/2000), most these cases were MSM [24]. The rapidly increasing trend of HIV infections among Hangzhou college students is alarming, as the number of reported HIV cases has increased from one in 2005, three in 2007, six in 2008, and to nineteen in 2009 [25]. According to a 2010 report from Chinese Ministry of Health, among nationally reported HIV/AIDS cases, the proportion of HIV among students increased from 0.96% to 1.04% to 1.37% in the years 2007, 2008 and 2009. During this time period (2007-2009), the proportion of HIV cases among 20-24 year old students increased from 20.3% to 39.8% and the proportion of homosexually transmitted cases increased from 8% to 36.9% [26].

Understanding the prevalence of HIV and high risk behaviour among Chinese student MSM is important in order to determine how to design interventions among this population to curb the HIV epidemic. The current study was conducted in order to determine HIV and syphilis prevalences, high risk sexual behaviours, drug use, and other factors related to HIV infection among Liaoning MSM students.

## Methods

### Participant enrolment

From April 2008 to January 2009, MSM students were approached and invited to participate in this study by outreach staff from non-governmental organizations that specifically serve the MSM community. Participants were eligible to participate in this study if they were male, at least 18 years of age, currently a high school or college student, reported at least one episode of receptive or insertive anal sex with a male sexual partner in the past 12 months, and were able and willing to provide written informed consent. Study participants were asked to refer their eligible sexual partners to participate in this study.

Data were collected on demographics, sexual behaviour, history of drug use (self-report of ever using opium, heroin, methamphetamine, morphine, cannabis, cocaine, dolantin, ketamine, triazolam, or amphetamine), and medical history through standardized questionnaires that were administrated though face-to-face interviews by trained local physicians. Blood specimens were obtained and tested for HIV and syphilis. Those who tested positive for HIV or syphilis were referred to treatment at a local hospital. All participants were given general information about HIV and syphilis and were informed how to practice safe sex through pre- and post-test counseling provided by this study. Each participant was compensated 50 RMB cash ($7 USD) or equivalent gifts as compensation for the cost of their time and transportation.

### Ethics

The study protocol and informed consent forms were approved by the Institutional Review Boards of the China Medical University and Liaoning Provincial Center for Disease Control and Prevention (CDC). Written informed consent was collected for each participant at enrolment. Participation in this study was completely voluntary and those who decided to participate could refuse to answer any questions they did not feel comfortable answering.

### Laboratory testing

Blood samples were tested for HIV and syphilis antibodies. The presence of HIV-1 antibody was tested for through enzyme-linked immunosorbent assay (ELISA) (Vironostika HIV-1 Microelisa System; BioMe´rieux, Durham, NC). Positive tests were confirmed by HIV-1/2 Western blot assay (HIV Blot 2.2 WB; Genelabs Diagnostics, Singapore). Syphilis serology was determined through rapid plasma reagin (RPR, Diagnosis t; Shanghai Kehua, China). Serum specimens that were positive for TPPA (TPPA, Serodia, Japan) were retested for rapid plasma reagin (RPR, Diagnosis t; Shanghai Kehua, China) assay, Subjects with serum positive for both TPPA and RPR were determined to be currently infected with syphilis. Syphilis tests and HIV antibody screening were conducted at local CDCs (Shenyang, Anshan, Fushun, Dalian and Benxi CDCs) and HIV WB confirmation was conducted at the Liaoning provincial CDC.

### Data analysis

Questionnaires were double entered and then checked for accuracy using Epi Data software (Epi Data Association, Odense, Denmark, version 3.02). Data were analyzed using SPSS version 16.0 (Chicago, IL, USA). Normally distributed continuous variables were described with means and non-normally distributed continuous variables were described with medians. Odds ratios (OR) and 95% confidence intervals (CI) were calculated. Categorical data were described and analyzed by frequency and Chi-squared tests. Univariate logistic regression and forward stepwise multivariate logistic regression were used to determine adjusted odds ratios (aOR) for HIV infection related risk-factors. Marginally significant variables with p < 0.20 in univariate analysis were included in multivariate analysis. Variables with p < 0.1 were retained in the final multivariate logistic model in a forward stepwise manner.

## Results

### Demographic characteristics of MSM students

A total of 436 eligible student MSM completed the survey. Demographic characteristics of study participants are shown in Table 1. The majority of study participants were ≤ 20 years of age (54.4%), single (98.2%), permanent residents of Liaoning Province (90.6%), Han ethnicity (88.5%), high school students (62.4%), identified as homosexual (57.8%), and met their sex partners on the internet (73.9%).

**Table 1:** Demographic characteristics of Liaoning MSM students

Characteristics	Number	Proportion (%)
Age (yrs.)		
≤ 20	237	54.4
> 20	199	45.6
Marriage status		
Single	428	98.2
Cohabitating	1	0.2
Married	7	1.6
Permanent residence		
Liaoning Province	395	90.6
Other province	41	9.4
Nationality		
Han	386	88.5
Non-Han	50	11.5
Currently level of education		
College or above	164	37.6
High school and below	272	62.4
Sexual orientation		
Homosexual orientation	252	57.8
Bisexual orientation	155	35.6
Heterosexual orientation	4	0.9
Sexual orientation undetermined	25	5.7
Main location to seek homosexual partners		
Internet	322	73.9
Park/public bathroom	35	8.0
Bathing room/sauna/massage room	27	6.2
Bar/night club/tearoom	26	6.0
Other places	26	6.0

### HIV-related knowledge

Table 2 illustrates the relatively high HIV-related knowledge exhibited by study participants. Approximately 90% knew the correct answers to HIV prevention questions, with the exception of the question about acquiring HIV through mosquito bites, which only 67.4% answered correctly.

**Table 2:** HIV/AIDS related knowledge among Liaoning MSM students (n = 436)

HIV/AIDS knowledge and prevention related questions	Correctly answered(%)
Whether PLWH can be detected from appearance	399(91.5%)
Whether behaviors can result in HIV infection	
Blood transfusion from PLWH	426(97.7%)
Sharing syringe with PLWH	414(95.0%)
Having dinner with PLWH	391(89.7%)
Birth and breast-feeding from HIV infected mothers	424(97.2%)
Mosquito bites	294(67.4%)
Limiting sexual partners to one single HIV-negative partner	399(91.5%)
Correctly using condom during each act of sexual intercourse	418(95.9%)

*, PLWH, people living with HIV;

### Drug use and high risk sexual behaviour

Only 13.5% (59/436) of MSM students' sexual orientation were known by their family, of which 10.8% (47/436) and 6.7% (29/436) were known by their mothers and fathers, respectively. The average age of first sexual intercourse was 18.5 ± 5.9 years, in which 428 (98.2%) had insertive anal intercourse with male sexual partners and 8 (1.8%) had vaginal intercourse with female partners. Approximately one-third (n = 146, 33.5%) had ever had an HIV test. In the most recent 6 months, 371(85.1%) had anal intercourse with male sexual partners. In the most recent 6 months, 57.1% (249/436) of participants had one male sexual partner, 28.7% (125/436) had 2-3 male sexual partners, 7.6% (33/436) had 4-5 male sexual partners, and 6.7% (29/436) had > 5 male sexual partners. Slightly more than half (n = 259, 59.4%) never used or seldom used condoms with male sexual partners in the most recent six months. When having anal sex with regular (non-casual) male sexual partners, only 180 (48.5%) always used a condom. Slightly more (n = 254, 58.3%) always used a condom when they had anal sex with occasional sexual partners. Most study participants did not use a condom during insertive oral sex (65.5%) or receptive oral sex (65.0%). One quarter (n = 104, 23.9%) experienced anal bleeding after receptive intercourse.

In the most recent six months, 7 (1.6%) purchased sex from male partners and 25 (5.7%) once sold sex to male partners. A small proportion of the study population (n = 43, 9.9%) had engaged in heterosexual behaviour, in which 21 (48.8%) failed to use condoms with female sexual partners. In the past 12 months, 55 (12.6%) study participants had sexually transmitted disease (STD) symptoms (pain during urination, genital burning sensation, or abnormal urethral discharge) in the past year, in which 98.2% (54/55) went to see a private doctor or self-treated.

Several study participants (n = 25, 5.7%) knew that their male sexual partners were infected with HIV, while 138 (31.7%) declined to used condoms with their regular male sexual partners even if they knew their regular male sexual partner was infected with HIV. Very few (n = 8, 1.8%) self-reported to have ever used any of the listed illegal drugs and none had ever injected drugs.

### HIV/syphilis prevalence and HIV infection correlates

The prevalence of HIV among Liaoning student MSM was 3.0% (13/436) (95% confidence interval (CI) 1.6%-5.0%). The prevalence of syphilis was 5.0% (22/436) (95% CI 3.2%-7.5%). Table 3 presents the univariate and multivariate logistic analyses of variables associated with HIV among Liaoning MSM students. In multivariate analysis, sexual orientation known by family members (OR: 7.3; 95% CI: 1.5-34.6), HIV/AIDS information obtained from clinical doctors (OR: 6.7; 95% CI: 1.7-25.9), HIV/AIDS information obtained through free educational services and materials such as voluntary counselling and testing (VCT) and condom distribution programs (OR: 0.2; 95% CI: 0.4-1.0), inconsistent condom use (OR: 5.7; 95%: 1.3-25.3), sexual partner experienced anal bleeding after insertive anal intercourse (OR: 6.8; 95% CI: 1.6-28.4), and history of illegal drug use (OR: 18.9; 95% CI: 2.2-165.3) were found to be significant associated for HIV infection.

**Table 3:** Prevalence, univariate, and multivariate logistic analyses of HIV infection associated factors among Liaoning MSM students

Factors		N	Prevalence (%)	Univariate Analysis	Multivariate Analysis	
				
				OR (95% CI)	AOR (95% CI)	P
Permanent residence	Liaoning Province	395	13(3.3)			
	None-Liaoning Province	41	0(0.0)	0.3 (0.0-5.8)		
Age (yrs.)	≤ 20	237	6(2.5)			
	> 20	199	7(3.5)	1.4 (0.5-4.2)		
Nationality	Han	386	12(3.1)			
	Non-Han	50	1(2.0)	1.6 (0.2-12.3)		
Currently level of education	College or above	272	7(2.6)			
	High school and below	164	6(3.7)	1.4 (0.5-4.3)		
Sexual orientation known by family members	No	377	9(2.4)			
	Yes	59	4(6.8)	3.0 (0.9-10.0)	7.3 (1.5-34.6)	0.013
Knowledge of whether PLWH can be detected from appearance	No	399	9(2.3)			
	Yes	26	4(15.4)	5.3 (1.5-18.0)		
Knowledge of whether input blood from PLWH can infect HIV	Yes	426	11(2.6)			
	No	10	2(20.0)	9.4 (1.8-50.0)		
Knowledge of whether sharing syringe with PLWH can infect HIV	Yes	414	11(2.7)			
	No	22	2(9.5)	3.7 (0.8-17.6)		
HIV/AIDS knowledge scores	≥8	244	7(2.9)			
	< 8	192	6(3.1)	1.1 (0.4-3.3)		
Source of HIV/AIDS knowledge	Yes	229	4(1.7)			
Television	No	207	9(4.3)	2.6 (0.8-8.4)		
Clinical doctors	No	358	7(2.0)			
	Yes	78	6(7.7)	4.2 (1.4-12.8)	6.7 (1.7-25.9)	0.006
Free education services and materials, such as VCT and provision of condoms	No	230	10(4.3)			
	Yes	206	3(1.5)	0.3 (0.1-1.2)	0.2 (0.4-1.0)	0.060
Condom use with casual male sexual partners	Always use	254	3(1.2)			
	Occasionally/never use	182	10(5.5)	4.9 (1. 3-17.9)	5.7 (1.3-25.3)	0.023
Unprotected receptive anal sex in past 6 months	No	203	4(2.0)	2.0 (0.6-6.7)		
	Yes	231	9(3.9)			
Anal bleeding after receptive anal intercourse	No	330	8(2.4)			

	Yes	104	5(4.8)	2.0 (0.7-6.4)		
Sexual partner experienced anal bleeding after insertive anal intercourse	No	360	7(1.9)			
	Yes	76	6(7.9)	4.3 (1.4-13.3)	6.8 (1.6-28.4)	0.009
Has ever purchased or sold sex with male partners	No	409	12(2.9)			
	Yes	27	1(3.7)	1.3 (0.2-10.2)		
History of illegal drug use	No	429	11(2.6)			
	Yes	8	2(25.0)	12.6 (3.3-69.8)	18.9 (2.2-165.3)	0.008
STD symptoms* in the past year	No	381	9(2.4)			
	Yes	55	4(7.3)	3.2 (1.0-10.9)		
Saw a private doctor in the past year	No	410	11(2.7)			
	Yes	26	2(7.7)	3.0 (0.6-14.4)		
Self-treated STD in the past year	No	397	10(2.5)			
	Yes	39	3(7.7)	3.2 (0.8-12.3)		
Had male sexual partners who are infected with HIV	No	411	11(2.7)			
	Yes	25	2(8.0)	3.2 (0.7-15.1)		
Syphilis	Negative	401	11(2.7)			
	Positive	22	2(9.1)			

*STD symptoms: urination pain, penis burning, or abnormal urethral discharge. OR, odds ratio; AOR, adjusted odds ratio; VCT, voluntary counseling and testing.

## Discussion

To our knowledge, this is the largest epidemiological study to estimate HIV and STD prevalence and describe the related risk behaviours among Chinese male high school and university student MSM. Understanding this population is important in order to design interventions to curb China's HIV epidemic.

The HIV prevalence among this group of Liaoning student MSM was 3.0%, which is over fifty times higher than the Chinese general population (0.057%) [3]. This prevalence is also higher than that reported among MSM students in Beijing (2.3%) [14] and Hefei(1.7%) [15]. The prevalence of syphilis in this survey (5.0%), however, is lower than that reported among MSM students in Beijing (7.43%) [14] and Hefei(7.4%) [15]. The prevalences of HIV and syphilis were markedly lower than that reported among MSM of all ages in Shenyang City, Liaoning Province (HIV 5.7% and syphilis 25.4%) [8].

Although the number of high school and college students is the largest in the world and 3.7%-10.3% of Chinese male college students report engaging in sexual activity with men [16,18], as yet, there have been few HIV/STD prevention programs that have targeted this vulnerable population. The relatively high prevalence of HIV and syphilis found in this survey demonstrates that it is urgent for the Chinese government to take action and target prevention interventions aimed at this group.

The current study also found a seemingly contradictory phenomenon, those MSM whose sexual orientation was known by family members were more likely to be infected with HIV, while a previous study of Chinese MSM in Hong Kong found that those who were open about their MSM status were more likely to use condoms [27]. It is possible that participants who were HIV positive were already aware of their status and had disclosed their status to their parents. Sexual orientation known by family could also be considered a proxy for openness of sexuality. Future studies should investigate the effects of social support and openness of sexuality on high risk behaviour among Chinese MSM.

Somewhat paradoxically, those who received HIV/AIDS education from a clinician were more likely to be infected with HIV than those who did not receive HIV education from a clinician. Due to the cross-sectional nature of this study, it is difficult to discern if HIV-related knowledge was gained prior to engaging in high risk behaviour. Those who had an STI and were more susceptible to HIV infection may be more likely to see a clinician and obtain HIV/STI prevention information. Overall, HIV/AIDS knowledge was not statistically correlated with HIV infection, which may indicate a low perception of risk. The utilization of free HIV education services and materials such as VCT and condoms distribution services, however, were negatively associated with HIV infection. Comprehensive interventions, which incorporate VCT and the provision of free condoms, should be considered since it appears as though HIV knowledge is not enough to prevent HIV among Chinese student MSM.

Inconsistent condom use with casual male sex partners was found to be independently associated for HIV infection. Low condom usage is of particular concern in this study population since several reported low condom use with female partners and with HIV positive partners. A study in Beijing also reported low condom use among college student MSM [14]. Peer education has been demonstrated to be an effective means of reaching both MSM and student populations to promote healthy behaviours, which may be used to reduce their risk of HIV infection [28-31].

This study also found that bleeding after insertive anal intercourse was associated with HIV infection. A study in Mexico City determined that MSM who bled after anal sex were more likely to be HIV positive compared with those that did not experience bleeding [32]. Ruptured mucous membranes that result from anal sex may facilitate the entry of the virus. MSM should be encouraged to use condoms with water-based lubricants during anal sex in order to minimize the risk of anal and rectal bleeding and general HIV/STD transmission.

The current study found that illegal drug use, but not injection drug use, was independently associated with HIV infection among MSM students. Injection drug use has historically been found to be a primary risk factor for HIV in China [33], but studies of MSM have also found use of non-injection drugs to be associated with HIV infection. These studies found that methamphetamine, amyl nitrate, and Viagra were the most common drugs associated with HIV infection [34,35]. The current study did not gather detailed information on types of drugs used, but future interventions should discourage illegal drug use, in general, in order to prevent high risk behaviour resulting from a loss of inhibition.

This study is subject to several limitations. This was a cross-sectional study and therefore temporal associations cannot be inferred. The survey asked questions about sensitive topics and participants may not have felt comfortable answering accurately. In order to minimize recall bias, questions were framed in terms of behaviour in the last 6 months; however, this may not be indicative of usual behaviour. Selection bias may have influenced the results of this study and since the survey subjects were a convenience sample of student MSM who live in Liaoning Province, the results of this study may not be generalizeable to the greater student MSM population. Since participants were asked to recruit their sexual partners there may have been some bias in the HIV and STI estimates. Although multivariate analysis was used to address possible confounding by study variables, it is possible that other confounding occurred by variables not measured in this study.

## Conclusions

The prevalence of HIV among MSM students has reached 3.0%, which is over fifty times higher than the Chinese general population. History of illegal drug use, but not injection drug use, was found independently associated with HIV infection. Approximately one-third (33.5%) had ever had an HIV test. Most MSM students have a good understanding of the means of HIV/AIDS transmission and prevention. However, high proportions of participants failed to use condoms with steady and casual male partners, putting them at high risk for HIV infection and transmission. Only about five percent of MSM students' sexual orientations were known by their parents. The findings indicate that MSM students are a vulnerable population for HIV infection in China. HIV screening should be prioritized for this group. Future interventions should discourage illegal drug use in order to prevent high risk behaviour resulting from a loss of inhibition. The sole strategy of HIV/AIDS knowledge promotion is not enough to prevent HIV epidemic among this population. Widespread family and school-based based HIV prevention strategies should be encouraged by utilizing China's mainstream media. This study's results have important significance for designing immediate and targeted interventions to curb HIV epidemic among this population.

## Competing interests

The authors declare that they have no competing interests.

## Authors' contributions

HS and CML participated in the design of the study protocol, JJX and KHR wrote the manuscript. MN and ZM supervised the implementation of the study. JJW, ZXC and KY performed the analysis of the data. All authors read and approved the final manuscript.

## Pre-publication history

The pre-publication history for this paper can be accessed here:

http://www.biomedcentral.com/1471-2458/11/287/prepub

## References

[B1] Donenberg GR Paikoff R Pequegnat W Introduction to the special section on families, youth, and HIV: Family-based intervention studies J Pediatr Psychol 2006 31 9 869 873 10.1093/jpepsy/jsj102 16467312

[B2] State Council AIDS Working Committee Office, China Ministry of Health, UN Theme Group on HIV/AIDS in China A Joint Assessment of HIV/AIDS Prevention, Treatment and Care in China 2007 Beijing, China: State Council AIDS Working Committee Office http://www.chinaids.org.cn 2010

[B3] Ministry of Health, People's Republic of China: 2009 estimates for the HIV/AIDS epidemic in China Ministry of Health, People's Republic of China, Joint United Nations Programme on HIV/AIDS 2010 World Health Organization http://www.chinaids.org.cn

[B4] Zhang D Bi P Changes in HIV prevalence and sexual behavior among men who have sex with men in a northern Chinese city: 2002-2006 J Infect 2007 55 5 456 463 10.1016/j.jinf.2007.06.015 17714786

[B5] Jia M Luo H Ma Y Wang N Smith K Mei J Lu R Lu J Fu L Zhang Q The HIV epidemic in Yunnan Province, China, 1989-2007 JAIDS 2010 53 Suppl S34 S40 20104107 10.1097/QAI.0b013e3181c7d6ff

[B6] Ma X Zhang Q He X Sun W Yue H Chen S Raymond HF Li Y Xu M Du H Trends in prevalence of HIV, syphilis, hepatitis C, hepatitis B, and sexual risk behavior among men who have sex with men: results of 3 consecutive respondent-driven sampling surveys in Beijing, 2004 through 2006 JAIDS 2007 45 5 581 587 10.1097/QAI.0b013e31811eadbc 17577125

[B7] Feng TJ Liu XL Cai YM Pan P Hong FC Jiang WN Zhou H Chen XS Prevalence of syphilis and human immunodeficiency virus infections among men who have sex with men in Shenzhen, China: 2005 to 2007 Sex Transm Dis 2008 35 12 1022 1024 18830135 10.1097/OLQ.0b013e31818600f4

[B8] Xu JJ Zhang M Brown K Reilly K Wang H Hu Q Ding H Chu Z Bice T Shang H Syphilis and HIV Seroconversion Among a 12-Month Prospective Cohort of Men Who Have Sex With Men in Shenyang, China Sex Transm Dis 2010 37 7 432 439 20375928 10.1097/OLQ.0b013e3181d13eed PMC5454492

[B9] Ruan Y Jia Y Zhang X Liang H Li Q Yang Y Li D Zhou Z Luo F Shi W Incidence of HIV-1, Syphilis, Hepatitis B, and Hepatitis C Virus Infections and Predictors Associated With Retention in a 12-Month Follow-Up Study Among Men Who Have Sex With Men in Beijing, China JAIDS 2009 52 5 604 610 10.1097/QAI.0b013e3181b31f5c 19710617

[B10] Ding Y Detels R Zhao Z Zhu Y Zhu G Zhang B Shen T Xue X HIV infection and sexually transmitted diseases in female commercial sex workers in China JAIDS 2005 38 3 314 319 15735451

[B11] Tian LG Ma ZE Ruan YH Cao XY Huang JP Wang DR Zhu GP Yao HM Han L Hao C Incidence rates of human immunodeficiency virus and syphilis as well as the rate of retention in a 6-month follow-up study of female sex workers in areas with heavy drug use in Xichang of Sichuan province, China Zhonghua Liu Xing Bing Xue Za Zhi 2006 27 11 939 942 17402192

[B12] Ruan Y Chen K Hong K He Y Liu S Zhou F Qin G Chen J Xing H Shao Y Community-based survey of HIV transmission modes among intravenous drug users in Sichuan, China Sex Transm Dis 2004 31 10 623 627 15389002 10.1097/01.olq.0000140018.24262.4a

[B13] Zhang Y Shan H Trizzino J Ruan Y Beauchamp G Māsse B Ma J Rui B Wang J Liu M HIV incidence, retention rate, and baseline predictors of HIV incidence and retention in a prospective cohort study of injection drug users in Xinjiang, China Int J Infect Dis 2007 11 4 318 323 10.1016/j.ijid.2006.09.001 17321184

[B14] Zheng JD Study on HIV high risk behaviors and their correlates among men who have sex with men in college students of Beijing 2008 Chi CDC

[B15] Zhu J Zhang H Wu H High risk sexual behavior and HIV/STD infection rate among 122 MSM from students Chin J AIDS STD 2007 13 4 350 352

[B16] Cong L Ono-Kihara M Xu G Ma Q Pan X Zhang D Homma T Kihara M The characterisation of sexual behaviour in Chinese male university students who have sex with other men: A cross-sectional study BMC Public Health 2008 8 1 250 10.1186/1471-2458-8-250 18647381 PMC2503976

[B17] The central government website Ministry of Education released the 2009 National Education Development Statistical Bulletin From China Ministry of Education 2010 Ministry of Education website http://www.gov.cn/gzdt/2010-08/03/content_1670245.htm 2011

[B18] XinHua Net Sexual condition survey of Chongqing college students: ten percent students had homosexual behavior Chongqing Moring newspaper 2010 http://news.xinhuanet.com/edu/2003-12/31/content_1255624.htm

[B19] Ruan Y Li D Li X Qian H Shi W Zhang X Yang Z Zhang X Wang C Liu Y Relationship between syphilis and HIV infections among men who have sex with men in Beijing, China Sex Transm Dis 2007 34 8 592 597 17325622 10.1097/01.olq.0000253336.64324.ef

[B20] Fleming DT Wasserheit JN From epidemiological synergy to public health policy and practice: the contribution of other sexually transmitted diseases to sexual transmission of HIV infection Sex Transm Infect 1999 75 3 17 10.1136/sti.75.1.3 10448335 PMC1758168

[B21] Wang S Wang L Qi H Rong X A Survey for the university students on HIV voluntary test and the awareness status of AIDS knowledge Chin J Health Educ 2007 23 6 447 478

[B22] Xu J Zhang H Zhang Y Wang J Zhu Y Li Z Hu Z The prevalence of syphilis and HIV infection among young men who have sex with men in Hefei city Chin J of Behavioral Med Sci 2007 16 3 205 207

[B23] Xu Z Ma S Wang R One HIV infection case report among MSM student Chin J Sch Health 2007 48 4 367 367

[B24] Global Network Zhejiang Province prepares to learn HIV prevalence among college students by physical examination 2010 http://china.huanqiu.com/roll/2010-07/919670.html

[B25] Zhejiang Online There is no high risk population but high risk behaviors: Face-to-face between students to AIDS patient China Daily 2010 http://www.chinadaily.com.cn/dfpd/zhejiang/2010-06-22/content_482573.html

[B26] Xinhua Net HIV epidemic is increasing among China aged population and campus students 2010 http://www.chain.net.cn/zhxw/xwbd/27868.htm

[B27] Wong CY Tang CS Sexual practices and psychosocial correlates of current condom use among Chinese gay men in Hong Kong Arch Sex Behav 2004 33 2 159 167 15146148 10.1023/b:aseb.0000014330.67201.1b

[B28] Boyer CB Sieverding J Siller J Gallaread A Chang YJ Youth united through health education: Community-level, peer-led outreach to increase awareness and improve noninvasive sexually transmitted infection screening in urban African American youth J Adolesc Health 2007 40 6 499 505 10.1016/j.jadohealth.2006.09.020 17531755

[B29] Kelly JA St Lawrence JS Diaz YE Stevenson LY Hauth AC Brasfield TL Kalichman SC Smith JE Andrew ME HIV risk behavior reduction following intervention with key opinion leaders of population: an experimental analysis Am J Public Health 1991 81 2 168 171 10.2105/AJPH.81.2.168 1990853 PMC1404968

[B30] Kelly JA St Lawrence JS Stevenson LY Hauth AC Kalichman SC Diaz YE Brasfield TL Koob JJ Morgan MG Community AIDS/HIV risk reduction: the effects of endorsements by popular people in three cities Am J Public Health 1992 82 11 1483 1489 10.2105/AJPH.82.11.1483 1443297 PMC1694607

[B31] Kelly JA Murphy DA Sikkema KJ McAuliffe TL Roffman RA Solomon LJ Winett RA Kalichman SC Randomised, controlled, community-level HIV-prevention intervention for sexual-risk behaviour among homosexual men in US cities J Lancet 1997 350 9090 1500 1505 10.1016/S0140-6736(97)07439-4 9388397

[B32] Coplan PM Gortmaker S Hemandez-Avila M Spiegelman D Uribe-Zuuiga P Mueller NE Human immunodeficiency virus infection in Mexico City: rectal bleeding and anal warts as risk factors among men reporting sex with men Am J Epidemiol 1996 144 9 817 827 8890660 10.1093/oxfordjournals.aje.a009016

[B33] Ma Y Li Z Zhang K HIV was first discovered among injection drug users in China Chin J Epidemiol 1990 11 184 185

[B34] Carey JW Mejia R Bingham T Ciesielski C Gelaude D Herbst JH Sinunu M Sey E Prachand N Jenkins RA Drug use, high-risk sex behaviors, and increased risk for recent HIV infection among men who have sex with men in Chicago and Los Angeles AIDS Behav 2009 13 6 1084 1096 10.1007/s10461-008-9403-3 18498049

[B35] Ostrow DG Plankey MW Cox C Li X Shoptaw S Jacobson LP Stall RC Specific sex drug combinations contribute to the majority of recent HIV seroconversions among MSM in the MACS JAIDS 2009 51 3 349 355 10.1097/QAI.0b013e3181a24b20 19387357 PMC3074969

